# Melanoma cells replicate through chemotherapy by reducing levels of key homologous recombination protein RAD51 and increasing expression of translesion synthesis DNA polymerase ζ

**DOI:** 10.1186/s12885-017-3864-6

**Published:** 2017-12-18

**Authors:** Liang Song, Ewan M. McNeil, Ann-Marie Ritchie, Katy R. Astell, Charlie Gourley, David W. Melton

**Affiliations:** 1 Edinburgh Cancer Research Centre, MRC Institute of Genetics and Molecular Medicine, University of Edinburgh, Western General Hospital, Crewe Road, Edinburgh, EH4 2XU UK; 2 Present Address: Centre for Neuroregeneration, University of Edinburgh, Chancellor’s Building, 49 Little France Crescent, Edinburgh, EH16 4SB UK

**Keywords:** Melanoma, Chemotherapy, Cisplatin, DNA repair, RAD51, Translesion synthesis, DNA polymerase zeta, Synthetic lethality, PARP inhibitor

## Abstract

**Background:**

The global incidence of melanoma has been increasing faster than any other form of cancer. New therapies offer exciting prospects for improved survival, but the development of resistance is a major problem and there remains a need for additional effective melanoma therapy. Platinum compounds, such as cisplatin, are the most effective chemotherapeutics for a number of major cancers, but are ineffective on metastatic melanoma. They cause monofunctional adducts and intrastrand crosslinks that are repaired by nucleotide excision repair, as well as the more toxic interstrand crosslinks that are repaired by a combination of nuclease activity and homologous recombination.

**Methods:**

We investigated the mechanism of melanoma resistance to cisplatin using a panel of melanoma and control cell lines. Cisplatin-induced changes in levels of the key homologous recombination protein RAD51 and compensatory changes in translesion synthesis DNA polymerases were identified by western blotting and qRT-PCR. Flow cytometry, immunofluorescence and western blotting were used to compare the cell cycle and DNA damage response and the induction of apoptosis in cisplatin-treated melanoma and control cells. Ectopic expression of a tagged form of RAD51 and siRNA knockdown of translesion synthesis DNA polymerase zeta were used to investigate the mechanism that allowed cisplatin-treated melanoma cells to continue to replicate.

**Results:**

We have identified and characterised a novel DNA damage response mechanism in melanoma. Instead of increasing levels of RAD51 on encountering cisplatin-induced interstrand crosslinks during replication, melanoma cells shut down RAD51 synthesis and instead boost levels of translesion synthesis DNA polymerase zeta to allow replication to proceed. This response also resulted in synthetic lethality to the PARP inhibitor olaparib.

**Conclusions:**

This unusual DNA damage response may be a more appropriate strategy for an aggressive and rapidly growing tumour like melanoma that enables it to better survive chemotherapy, but also results in increased sensitivity of cultured melanoma cells to the PARP inhibitor olaparib.

**Electronic supplementary material:**

The online version of this article (10.1186/s12885-017-3864-6) contains supplementary material, which is available to authorized users.

## Background

Over the last 30 years the global incidence of melanoma has increased faster than any other form of cancer. Global incidence in 2012 was 232,000, and is predicted to increase to 280,000 by 2020 [1]. It is now the second most common cancer amongst young adults in the UK [2]. Early surgical removal of primary tumours is an effective treatment, but up to 20% of patients may go on to develop metastatic disease, which has a very poor prognosis. Standard chemotherapeutics, that have a proven record of success against many different cancers, are ineffective on metastatic melanoma. Five year survival with the standard treatment, the alkylating agent dacarbazine is <15% [3]. Global mortality in 2012 was 55,500, and is predicted to increase to 67,800 by 2020 [1]. The new BRAF inhibitors, combination therapies and immunotherapies offer exciting prospects for improved survival, but not all patients respond and the development of resistance is a major problem [4, 5], hence there remains a need for additional effective melanoma therapy.

One possible approach is the use of PARP inhibitors, such as olaparib. They are most effective as anticancer agents in situations where the homologous recombination repair (HRR) pathway for the repair of dsDNA breaks is defective. Olaparib causes PARP to be trapped onto DNA repair intermediates, especially during base excision repair. This may cause obstruction to replication forks that is normally resolved by BRCA-dependent homologous recombination. If this pathway is non-functional then apoptosis is triggered [6]. The paradigm for this synthetic lethality is in BRCA-mutant ovarian and breast cancers and it is to these cancers that PARP inhibitors have initially been targeted [7–10]. A number of genes in addition to BRCA1 and 2 are involved in the HRR pathway and a mutation in any one of these could also lead to synthetic lethality with olaparib. This realisation has triggered a search to identify other cancer patients with a “BRCAness” phenotype that should also respond to PARP inhibitor therapy. One biomarker suggested to monitor the activity of the HRR pathway is RAD51, the enzyme responsible for the key strand exchange step in the process. In principle this synthetic lethality approach should also be applicable to melanoma. However, one approach, combining olaparib with dacarbazine, found no clinical advantage over dacarbazine alone [11].

Increased expression of many DNA repair genes has been found in primary melanomas that went on to metastasize compared to non-recurrent primaries [12–14]. We have been investigating the possibility that alterations in DNA repair gene expression could contribute to the extreme resistance that melanoma shows to chemotherapy. Platinum compounds, such as cisplatin, are the most effective chemotherapeutics for a number of major cancers, such as ovarian and lung. They cause monofunctional adducts and intrastrand crosslinks that are repaired by the nucleotide excision repair (NER) pathway, and also the more toxic interstrand crosslinks (ICLs) that are repaired by a combination of nuclease activity and HRR [15]. The ERCC1-XPF endonuclease is required for both repair pathways [16]. We have previously shown that ERCC1 and XPF gene expression is elevated by exposure of melanoma cells to cisplatin and that ERCC1 inactivation or inhibition renders melanoma sensitive to cisplatin [17–19].

Here we identify and characterise a novel DNA damage response mechanism in melanoma, involving inactivation of the key HRR protein RAD51 and increased translesion synthesis DNA polymerase activity, that allows melanoma cells to continue to grow in the presence of cisplatin. We also test the hypothesis that this response might also render melanoma susceptible to synthetic lethality with olaparib.

## Methods

### Mammalian cells

Human melanoma cells A375, C32, G361, HBL and WM115, and MRC5v1 SV40 transformed primary human fibroblasts [20], all authenticated by short tandem repeat profiling, were obtained from The European Collection of Cell Cultures (Salisbury, UK). Early passage established human ovarian cancer cell lines, PEO1 and PEO4, were kindly provided by their isolator Dr. Simon Langdon, University of Edinburgh, directly from frozen stocks established soon after their original isolation [21]. All experiments were performed on cultures within 10 passages of their supply. Cells were maintained in DMEM medium (41965; Life Technologies Ltd., Paisley, UK), or RPMI 1640 medium (PEO1 and 4 only; 21875, Life Technologies Ltd.), supplemented with 10% FCS, Non-Essential Amino Acids (11140–035; Life Technologies Ltd.), 1 mM Sodium Pyruvate, 2 mM L-glutamine and Penicillin [100 U/ml] –Streptomycin (100 mg/ml) at 37 °C, 5% CO_2_.

### Platinum response assays

A375 cells were plated at 55000 cells/well in 6-well plates. Twenty-four hours later cells were transfected using Lipofectamine 2000 as recommended by the supplier (Life Technologies Ltd.). Plasmid pCMV5 FLAG-RAD51 (90 ng, supplied by MRC Protein Phosphorylation and Ubiquitylation Unit, University of Dundee, UK) was used/well to express equivalent levels of FLAG-RAD51 to endogenous RAD51. In separate transfections 40 pmol of ON-TARGETplus REV3L (5980) siRNA SMARTpool/well, with ON-TARGETplus Non-targeting Pool control siRNA (GE Dharmacon, Lafayette, CO), was used to knockdown DNA Pol ζ. Four hours after transfection 1 μM cisplatin (Hospira UK Ltd., Leamington Spa, UK) was added to some transfected and non-transfected wells. Cells were harvested 24, 48 and 72 h later for cell number counts, flow cytometry and protein lysates. Flow cytometry, to determine the effect of cisplatin on cell cycle status, was carried out by propidium iodide staining as described [22].

### Cell culture toxicity assays

A375 cells were plated at 500 cells per well directly into 96-well plates, containing an olaparib dilution series, in the presence or absence of cisplatin. DMSO was maintained at <0.75%. Plates were cultured for 5 days and cell growth was quantified using a Sulphorhodamine B (SRB) assay [23].

### Western blotting

Protein extraction was carried out on ice using RIPA buffer (25 mM Tris-HCl pH 7.2, 150 mM NaCl, 1% Triton X-100, 1% deoxycholate, 1 mM EDTA, 20 mM NaF, 100 μM orthovanadate), with Roche complete protease inhibitor cocktail (Roche Products Ltd., Welwyn Garden City, UK). Primary antibodies: ERCC1, rabbit polyclonal FL-297 at 1:1000 dilution (Santa Cruz Biotechnology Inc., Dallas, TX); XPF, mouse monoclonal Ab-5 at 1:1000 (Thermo Fisher Scientific UK Ltd., Loughborough, UK); XPA, rabbit polyclonal NB100–92124 at 1:1000 (Novus Biologicals LLC., Littleton, CO); RAD51, rabbit polyclonal PC130 at 1:1000 (Merck Millipore Corporation, Darmstadt, Germany); DNA pol ζ, goat polyclonal sc-5939 at 1:100 (Santa Cruz Biotechnology Inc.); DNA pol η, rabbit polyclonal ab180703 at 1:200 (Abcam, Cambridge, UK); FLAG tag, mouse monoclonal F1804 at 1:1000 (Sigma-Aldrich, Poole, UK); cleaved Caspase-3, rabbit polyclonal #9661 at 1:1000 (Cell Signaling Technology, Danvers, MA); β-actin, mouse monoclonal A1978 at 1:10,000 (Sigma-Aldrich, Poole, UK), Lamin A/C, rabbit polyclonal #2032 at 1:1000 (Cell Signaling Technology). Secondary antibodies used following the primary antibody incubations: HRP-conjugated forms of goat anti-rabbit (P0448 at 1:3000, DAKO UK Ltd., Ely, UK), rabbit anti-goat (P0449 1:4000, DAKO) and rabbit anti-mouse (P0260 1:2000, DAKO). Western blotting was performed as described [18].

### Quantitative RT-PCR

Total RNA was extracted and quantitative RT-PCR reactions were carried out as described [17]. The primer pairs used were: q-PCR TaqMan RAD51 primer pair Hs00153418, q-PCR TaqMan beta-actin primer pair 4352935E.

### Determination of RAD51 DNA repair foci

Cells were seeded onto coverslips in wells of a 6-well plate containing 2 ml of medium and incubated at 37 °C for 24 h to allow them to adhere. They were then incubated for a further 24 h in control medium or medium with 6 μM cisplatin before fixing in 4% paraformaldehyde at 37 °C for 15 min. Cells were then washed 3 times with PBS for 5 min with gentle agitation, permeabilised with methanol:acetone (1:1) at −20 °C for 5 min, followed by another 3 PBS washes and stored in PBS at 4 °C until required. For RAD51 immunofluorescence, after 60 min blocking with 10% donkey serum, rabbit polyclonal antibody PC130 (1:400 dilution) was used overnight at 4 °C. Following 3 PBS washes, coverslips were incubated with fluorochrome-conjugated secondary antibody diluted 1:1000 for 60 min at room temperature (Alexa Fluor® 488 AffiniPure Donkey Anti-Rabbit IgG (H + L) 711–545-152; Jackson ImmunoResearch Laboratories, Inc., West Grove, PA). Coverslips were then washed and dried overnight in the dark before being mounted in VECTASHIELD Mounting Medium with DAPI (H-1200, VECTOR Laboratories Ltd., Peterborough, UK). Hardware control, immunofluorescence image capture and analysis were performed using Volocity 3D image analysis software (PerkinElmer Inc., Waltham, MA).

## Results

### Decreased RAD51 levels in A375 melanoma cells after cisplatin treatment

**Fig. 1:**
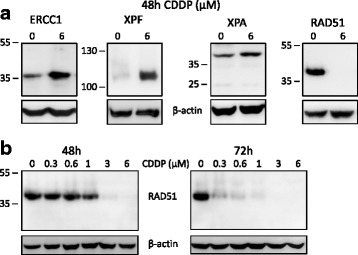
Reduced RAD51 levels in melanoma cells after cisplatin treatment. **a** Levels of Nucleotide Excision Repair proteins increase after cisplatin treatment of A375 melanoma cells, while levels of RAD51 decrease dramatically. Total protein extracts from untreated A375 human melanoma cells and cells treated with 6 μM cisplatin (CDDP) for 48 h were western blotted for the NER proteins, ERCC1 (33 kDa), XPF (104 kDa) and XPA (40 kDa) and the homologous recombination protein, RAD51 (37 kDa). β-actin (42 kDa) served as the loading control. **b** Cisplatin concentration- and treatment time-dependent decrease in RAD51 levels in A375 melanoma cells. Total protein extracts from untreated A375 human melanoma cells and cells treated with CDDP concentrations ranging from 0.3 to 6 μM for 48 and 72 h were western blotted for RAD51. β-actin served as the loading control. The positions of molecular weight markers (in kDa) adjacent to proteins of interest are shown

When A375 human melanoma cells were treated with 6 μM cisplatin for 48 h, an increase in the level of three NER proteins, ERCC1, XPF and XPA, was seen. However, when the same samples were probed for RAD51, we observed a dramatic and unexpected decrease in the level of this key HR protein that is needed for the removal of cisplatin-induced ICLs by HRR (Fig. 1a). We then investigated the time course and concentration-dependence of this RAD51 response to cisplatin. After 48 h of treatment the decrease was detectable at 1 μM cisplatin and, by 72 h, the level of RAD51 was much reduced at the lowest cisplatin concentration used, 0.3 μM (Fig. 1b). After 72 h of treatment at the highest cisplatin concentrations (3 and 6 μM), RAD51 protein was barely detectable by western blot.

### Decreased RAD51 levels after cisplatin treatment is particular to melanoma cells

**Fig. 2:**
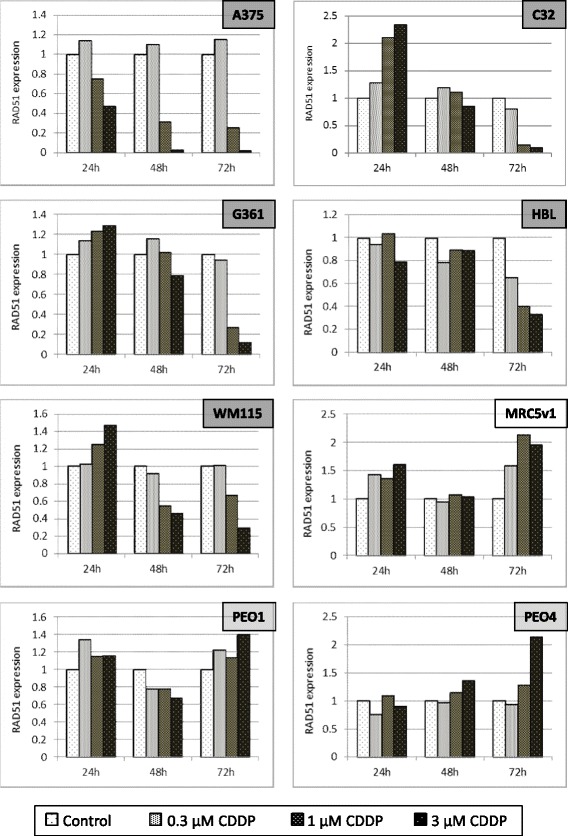
The reduction in RAD51 levels after cisplatin treatment in melanoma cells is not seen in other cell types. Graphs show the expression of RAD51, determined by western blotting, in five human melanoma cell lines (A375, C32, G361, HBL and WM115), two human ovarian cancer cell lines (PEO1 and PEO4) and the immortalised human fibroblast line, MRC5v1. Cells were untreated, or treated with 0.3, 1 or 3 μM cisplatin for 24, 48 or 72 h. RAD51 expression is plotted relative to β-actin and normalised to the level in untreated cells. Results are the means from two separate determinations. Representative western blots themselves are shown in Additional file 1: Figure S1

To investigate whether this unexpected RAD51 response to cisplatin was peculiar to A375 cells, or was instead a more general feature of melanoma, we assembled a panel of metastatic (A375, G361, HBL) and primary (C32 and WM115) human melanoma cell lines for further study. For comparison we also included an immortalised human fibroblast line (MRC5v1) and two human high grade serous ovarian cancer cell lines. PEO1 was isolated from a patient with a platinum-sensitive cancer, PEO4 was isolated from the same patient after the cancer became platinum-resistant. PEO1 is a BRCA2 mutant, PEO4 is a BRCA2 revertant. Cultures were treated with 0.3, 1 and 3 μM cisplatin for 24, 48 and 72 h. Although there was some apoptosis evident in dishes treated with 3 μM cisplatin for 72 h, cultures from all cell lines remained alive and healthy for the duration of this experiment. Cell cycle analysis and apoptosis in cisplatin-treated cultures are described later in the Results section. Representative western blots for RAD51 and the β-actin loading control are shown in Additional file 1: Figure S1 and histograms of RAD51 protein expression relative to β-actin and normalised to the level in untreated control cultures are shown in Fig. 2. Although there was some heterogeneity in the timing of the RAD51 response of the melanoma cell lines, with C32 in particular showing an initial 2-fold increase at 24 h with the higher cisplatin concentrations, all showed the same major reduction in RAD51 levels seen previously in A375 after 72 h of 1 and 3 μM cisplatin treatment (>65% reduction at 3 μM). The situation was very different in the non-melanoma lines at the same time point, where none showed any RAD51 reduction and, for MRC5v1 and PEO4, increased RAD51 protein expression after 72 h of 1 and 3 μM cisplatin treatment was evident. A similar increase in RAD51 levels after cisplatin treatment has been reported in lung cancer cell lines [24].

### No increase in RAD51 DNA repair foci in the short term response of melanoma cells to cisplatin

**Fig. 3:**
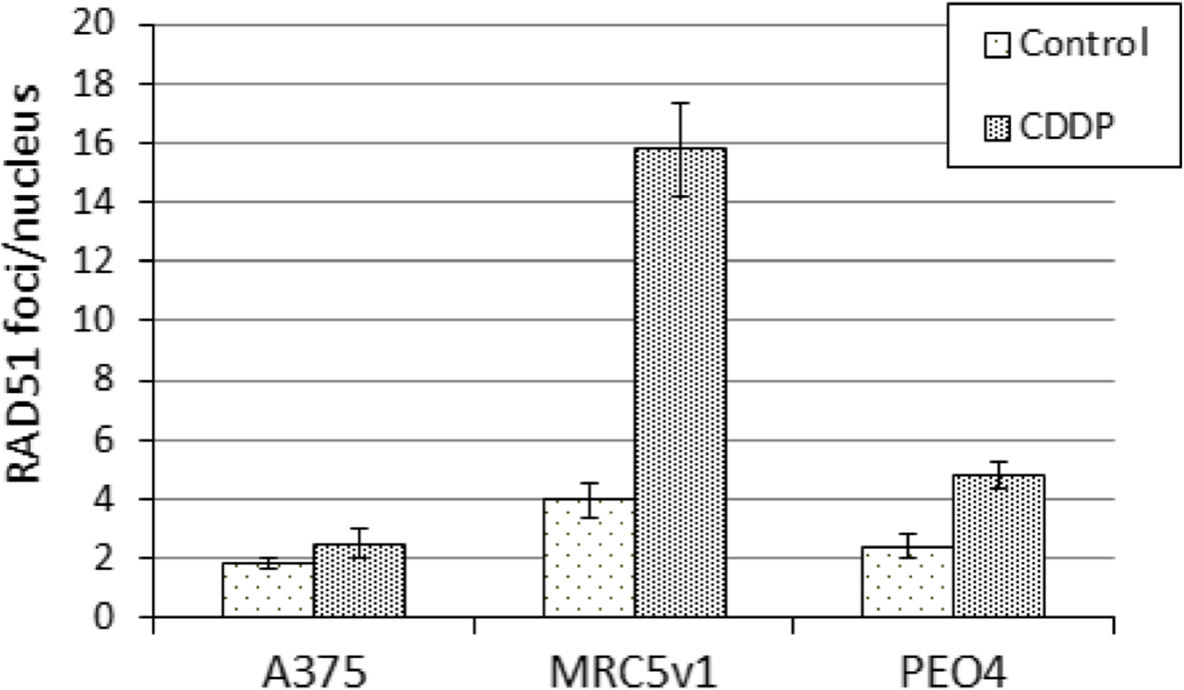
RAD51 DNA repair foci are not induced by short term cisplatin treatment of melanoma cells. A375 melanoma, MRC5v1 immortalised fibroblasts and PEO4 ovarian cancer cells growing on coverslips were treated for 24 h with 6 μM cisplatin and the number of RAD51 foci in treated and control cultures was determined by RAD51 immunofluorescence of fixed cells. Graph shows the mean number of RAD51 foci per nucleus (±SEM) for treated and control cultures. Between 40 and 60 nuclei were scored for each cell type and treatment. Representative images are shown in Additional file 1: Figure S2

RAD51 protein levels in A375 melanoma cells had already reduced by 50% after 24 h of 3 μM cisplatin treatment, with no equivalent reduction seen in non-melanoma cells (Fig. 2). To investigate the DNA repair response during this period we used immunofluorescence to investigate RAD51 DNA repair foci following 24 h of 6 μM cisplatin treatment in A375 melanoma, PEO4 ovarian cancer cells and MRC5v1 immortalised fibroblasts. The results are shown in Fig. 3 and representative images are in Additional file 1: Figure S2. MRC5v1 showed the classic repair response to cisplatin-induced DNA damage, with a significant 4-fold increase (*P* = <0.001 by Student’s t test) in the number of RAD51 foci in cisplatin-treated cells compared to controls. The BRCA2 revertant ovarian cancer cell line, PEO4, showed a smaller but still significant (*P* = 0.001) 2-fold increase in RAD51 foci after cisplatin treatment. The response of A375 melanoma cells was very different with no significant (*P* = 0.44) increase in RAD51 foci after cisplatin. We conclude that the reduction in RAD51 levels in A375 melanoma cells on cisplatin treatment is accompanied by a different DNA repair response to that seen in the non-melanoma cells.

### The fall in RAD51 protein in cisplatin-treated melanoma cells is due to reduced RAD51 mRNA levels

**Fig. 4:**
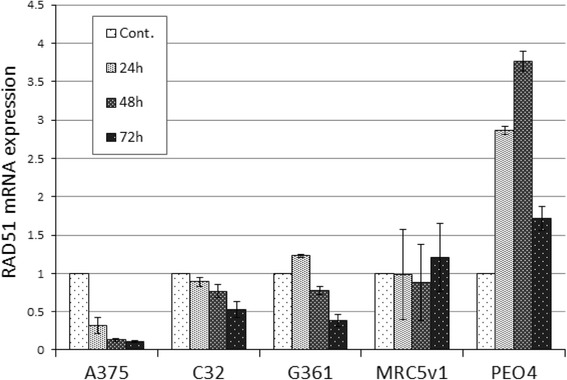
Reduced RAD51 mRNA levels in melanoma cells after cisplatin treatment. A375, C32, G361 melanoma cells, PEO4 ovarian cancer cells and MRC5v1 fibroblasts were treated with 3 μM cisplatin for 24, 48 or 72 h. RNA was extracted and RAD51 mRNA levels were determined by quantitative RT-PCR. RAD51 mRNA expression (±SEM, *n* = 3) is shown relative to β-actin and normalised to the level in untreated control (Cont.) cells

To investigate the basis for the reduced RAD51 levels after cisplatin treatment of melanoma cells, cultures of A375, C32, G361, PEO4 and MRC5v1 cells were treated with 3 μM cisplatin for 24, 48 and 72 h before RNA was extracted and RAD51 mRNA levels were determined by quantitative RT-PCR. Levels of RAD51 mRNA, relative to β-actin in cisplatin-treated cultures and normalised to the level in control cultures, are shown in Fig. 4. RAD51 mRNA levels in A375 were already reduced to 32% of the untreated control after 24 h of cisplatin treatment, with levels falling further with prolonged treatment, down to 11% at 72 h. RAD51 mRNA levels also fell progressively with cisplatin treatment in the other two melanoma cell lines examined, C32 and G361, albeit not to the same extent as seen in A375. No similar reduction in RAD51 mRNA levels was seen in PEO4 and MRC5v1, instead levels were unaltered over the time course of cisplatin treatment in MRC5v1 and rose by up to 3.8-fold in PEO4. We next investigated whether increased proteolysis by either of the two main pathways was also contributing to the cisplatin-induced reduction in RAD51 levels in melanoma. Proteasome-dependent proteolysis was inhibited with MG132 or bortezomib, while bafilomycin A1 was used to block the lysosome-dependent pathway. A375 and C32 cells treated with 3 μM cisplatin for 72 h were also exposed in combination with MG132 (1 μM) (Additional file 1: Figure S3A). There was no evidence that blocking proteasome-mediated proteolysis could reverse the cisplatin-induced reduction of RAD51 levels. As a positive control for inhibition of proteasomal degradation, A375 cells treated with MG132 showed an ~2-fold increase over control cells in levels of the nucleotide excision repair protein ERCC1 (Additional file 1: Figure S3B), which we have previously shown to be degraded by the polyubiquitination-dependent proteasome pathway [25]. Similarly, treatment of A375 cells with proteasome inhibitor bortezomib (10 nM) was unable to reverse the cisplatin-induced reduction in RAD51 levels (Additional file 1: Figure S3C). Lysosome-dependent proteolysis inhibitor bafilomycin A1 (1 μM) also had no effect on RAD51 levels in cisplatin treated G361 cells (Additional file 1: Figure S3D). Although there were some discrepancies between RAD51 protein and mRNA levels, we conclude that the cisplatin-induced reduction in RAD51 levels in melanoma cells is due to reduced RAD51 mRNA levels rather than increased proteolysis.

### The cisplatin-induced reduction in RAD51 induced synthetic lethality to olaparib in A375 melanoma cells

**Fig. 5:**
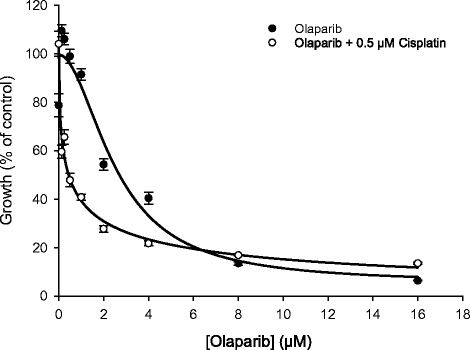
Cisplatin enhances the sensitivity of melanoma cells to PARP inhibitor olaparib. Five-day SRB growth assay on A375 melanoma cells in 96-well plates showing enhanced sensitivity to olaparib in the presence of 0.5 μM cisplatin. This cisplatin concentration is well below the IC50 value. In order to correct for the toxicity of cisplatin alone, for each curve growth is expressed as the percentage of the non-olaparib-treated control. Values plotted are mean % growth (±SEM) from three independent experiments

The unusual RAD51 response to cisplatin in melanoma cells led us to hypothesise that we could induce synthetic lethality and so render melanoma sensitive to PARP inhibitors by simultaneous exposure to cisplatin. The result of an A375 growth assay in a dilution series of olaparib in the presence and absence of 0.5 μM cisplatin is shown in Fig. 5. This cisplatin concentration is well below the IC50 value (1.04 μM) in the 5-day growth assay that we have reported previously [19]. When the toxicity of cisplatin alone was corrected for, we found that cisplatin caused a modest 5-fold enhanced sensitivity to olaparib (olaparib IC50 2.56 μM, olaparib plus cisplatin IC50 0.45 μM, *P* < 0.0001). Our other melanoma lines were not tested for induced synthetic lethality to olaparib.

### Melanoma cells show only a weak G2 arrest after cisplatin treatment

We next began to consider how the unusual RAD51 response of melanoma cells to cisplatin might be connected to the resistance of melanoma to chemotherapy. A strong G2 arrest, triggered by DNA replication arrest at interstrand crosslinks, is the normal response to cisplatin. The cell cycle profiles of A375 melanoma, MRC5v1 fibroblasts and platinum sensitive, PEO1, and resistant, PEO4, ovarian cancer cells were determined in response to 0.3 and 1 μM cisplatin treatment for 24 and 48 h (Additional file 1: Figure S4).The A375 profile was unaffected by 0.3 μM cisplatin, while at 1 μM there was a weak accumulation of cells in G2 at 24 h, which had resolved by 48 h. PEO1 showed an accumulation of cells in G2 at the lowest cisplatin concentration, while PEO1, PEO4 and MRC5v1 showed strong G2 arrests at 1 μM cisplatin.

### The RAD51 response of melanoma cells to cisplatin is a specific transcriptional response resulting in improved survival and growth

**Fig. 6:**
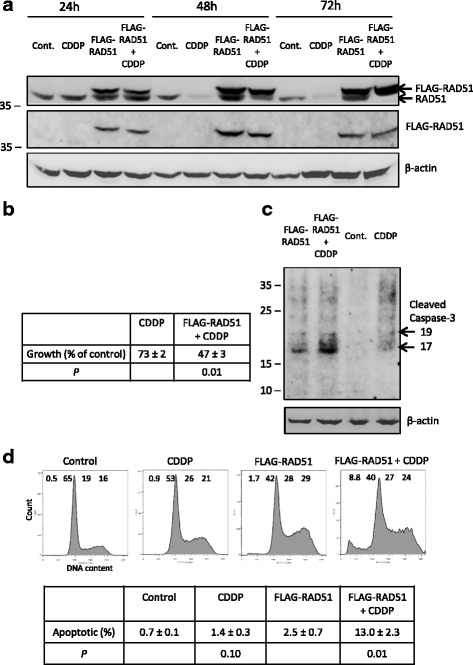
Expression of FLAG-tagged RAD51 adversely affects the response of melanoma cells to cisplatin. **a** No cisplatin-induced reduction in levels of FLAG-RAD51. Western blots from control A375 cells, cells transfected to express FLAG-RAD51 at levels equivalent to endogenous RAD51 and cells of both types treated with 1 μM cisplatin for 24, 48 or 72 h. The blot was probed sequentially for RAD51 (RAD51 37 kDa, FLAG-RAD51 38.2 kDa), the FLAG tag, and β-actin (42 kDa). **b** Reduced growth in cisplatin-treated cultures expressing FLAG-RAD51. The table shows the mean cell growth (±SEM, *n* = 4) of wells after 72 h of 1 μM cisplatin treatment for non-transfected A375 cells expressed as a percentage of the untreated control, and for FLAG-RAD51 transfected A375 cells expressed as a percentage of the non-cisplatin-treated FLAG-RAD51 transfection control. The *P* value for Student’s paired t test, comparing the effect of cisplatin on growth of normal and FLAG-RAD51-expressing cells is also shown. **c** Increased early apoptosis in cisplatin-treated A375 cultures expressing FLAG-RAD51. Cultures treated as in (**a**) and western blotted for activated caspase-3 and β-actin after 48 h of 1 μM cisplatin treatment. Note the highest levels of cleaved caspase-3 (at 17 and 19 kDa) in the sample transfected with FLAG-RAD51 and treated with cisplatin. **d** Increased apoptosis in cisplatin-treated A375 cells expressing FLAG-RAD51. Cultures treated as in (**a**) after 72 h of 1 μM cisplatin. A representative flow cytometry profile for each culture condition is shown, with the percentage of cells with subG1 (apoptotic), G1, S and G2/M DNA contents indicated across the top of each profile. Note the highest level of subG1 (apoptotic) material in the cisplatin-treated FLAG-RAD51-expressing cells. The table below shows the mean level of apoptosis (±SEM, *n* = 4) in wells after 72 h of cisplatin treatment. The *P* values for Student’s paired t test, comparing the level of apoptosis between cisplatin-treated and untreated control wells, and between cisplatin-treated FLAG-RAD51-expressing wells and untreated FLAG-RAD51-expressing wells are also shown

Next we investigated the significance and specificity of the RAD51 response of melanoma cells to cisplatin by expressing an N-terminal FLAG-tagged version of RAD51, under vector rather than endogenous RAD51 promoter control, at levels equivalent to those of the endogenous RAD51 protein. This tag has been used extensively and is considered unlikely to affect the intracellular location, stability and function of the tagged protein [26]. If the RAD51 response was merely a non-specific consequence of cisplatin treatment, for instance as a result of apoptosis-induced proteolysis, then both endogenous and FLAG-RAD51 proteins would be expected to respond in the same way. This was not what we observed (Fig. 6a). Endogenous RAD51 protein showed the expected dramatic reduction over the 72 h exposure to 1 μM cisplatin, while expression of FLAG-RAD51 was essentially unaffected, thus indicating the transcriptional specificity of the RAD51 response to cisplatin.

When untreated control A375 wells were harvested at the same time as wells treated for 72 h with 1 μM cisplatin, the mean cell number in control wells had increased 55-fold since plating. Untreated FLAG-RAD51-expressing cells showed slower growth, with a 20-fold increase in cell number over the same period and a moderately elevated level of apoptosis (2.5% compared to 0.7% for control cells, Fig. 6d). The mean cell number of 1 μM cisplatin-treated cultures was 73 ± 2% of the untreated control, while the mean cell number of the cisplatin-treated cultures expressing FLAG-RAD51 was significantly less (*P* = 0.01 by Student’s t test), at only 47 ± 3% of the untreated FLAG-RAD51-expressing control (Fig. 6b). Activation (cleavage) of caspase-3 is one of the key early steps in apoptosis. Wells harvested after 48 h were also western blotted with an antibody specific for the cleaved forms of caspase-3 (Fig. 6c). Although cleaved caspase-3 (17 and 19 kDa fragments) were present in untreated FLAG-RAD51-expressing cells, the highest levels were found in cisplatin-treated FLAG-RAD51-expressing cells, where the cleaved caspase-3/ β-actin ratio was 2.5-fold higher than the non-cisplatin-treated FLAG-RAD51-expressing control. Representative cell cycle profiles of control, cisplatin-treated, FLAG-RAD51-expressing, and cisplatin-treated FLAG-RAD51-expressing cultures after 72 h are shown in Fig. 6d. The small increase in the percentage of cells with sub-G1 DNA content (apoptotic) between cisplatin-treated and control cultures was not significant, but the 5-fold increase in apoptosis between cisplatin-treated FLAG-RAD51-expressing and untreated FLAG-RAD51-expressing controls was significant (*P* = 0.01 by Student’s t test, *n* = 4). We conclude that the novel RAD51 transcriptional response to cisplatin in melanoma cells results in improved survival and growth over the time course of our assay compared to melanoma cells also expressing near endogenous levels of RAD51, but under ectopic promoter control.

### Melanoma cells respond to cisplatin with elevated levels of translesion synthesis DNA polymerase zeta resulting in improved survival and growth

**Fig. 7:**
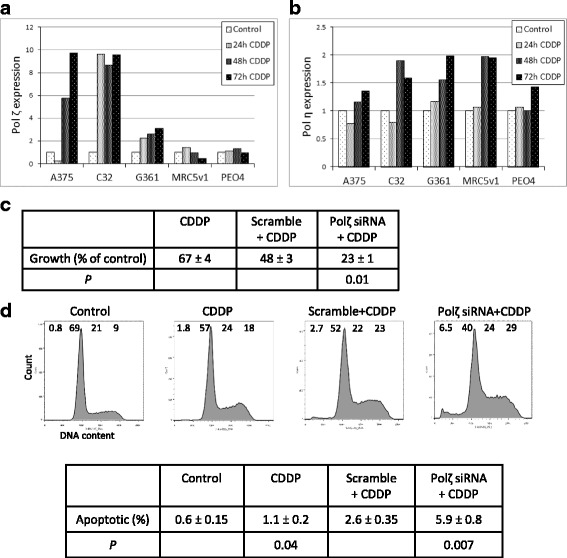
Increased levels of translesion synthesis DNA Polymerase zeta in melanoma cells improves their response to cisplatin. **a** and **b** Increased levels of DNA Polymerase zeta, but not DNA Polymerase eta after cisplatin treatment. Melanoma cell lines (A375, C32, G361), ovarian cancer cell line, PEO4, and immortalised fibroblast line, MRC5v1, were untreated, or treated with 3 μM cisplatin for 24, 48 or 72 h and western blotted for translesion synthesis DNA Polymerases. **a** DNA Pol ζ. **b** DNA Pol η. Graphs shows the expression relative to β-actin and normalised to the level in untreated cells. Results are the means from two separate determinations. Representative western blots themselves are shown in Additional file 1: Figure S6. **c** Reduced growth in cisplatin-treated cultures following siRNA knockdown of DNA Polymerase zeta. The table shows the mean cell growth (±SEM, *n* = 4) of wells after 72 h of 1 μM cisplatin treatment for non-transfected A375 cells and cells transfected with DNA Polymerase zeta siRNA or scrambled control siRNA, expressed as a percentage of the untreated control. The *P* value for Student’s paired t test, comparing the effect of DNA Polymerase zeta siRNA or scrambled control siRNA transfection on cell growth of cisplatin-treated wells is also shown. **d** Increased apoptosis in cisplatin-treated DNA Pol ζ siRNA-transfected A375 cells. Cultures treated as in (**c**) after 72 h of 1 μM cisplatin. A representative flow cytometry profile for each culture condition is shown, with the percentage of cells with subG1 (apoptotic), G1, S and G2/M DNA contents indicated across the top of each profile. Note the increased level of subG1 (apoptotic) material in the cisplatin-treated DNA Pol ζ siRNA-transfected cells. The table below shows the mean level of apoptosis (±SEM, *n* = 4) in wells after 72 h of cisplatin treatment. The *P* values for Student’s paired t test, comparing the level of apoptosis between cisplatin-treated and untreated control wells, and between cisplatin-treated DNA Pol ζ siRNA-transfected wells and cisplatin-treated scrambled control siRNA-transfected wells are also shown

How are melanoma cells able to continue to cycle in the presence of 1 μM cisplatin? We have shown previously [17], and again here, that levels of some NER proteins are elevated in response to cisplatin. This could make melanoma cells better able to repair cisplatin-induced bulky adducts and intrastrand crosslinks using NER. Increased levels of ERCC1-XPF endonuclease activity could also facilitate increased repair of cisplatin-induced ICLs by HRR during S phase, but melanoma cells show strong transcriptional downregulation of the key HRR protein RAD51 in response to cisplatin? Translesion synthesis (TLS) DNA polymerases are able to insert bases on a damaged template to allow replication to proceed. Since two such polymerases, DNA pol ζ (zeta) and DNA pol η (eta), have been implicated in cisplatin bypass [27, 28], we decided to investigate whether the novel RAD51 response of melanoma to cisplatin also involved altered expression of these two TLS polymerases. DNA Pol ζ proved a difficult target to detect by western blotting, with the large catalytic subunit (REV3L) at 353 kDa and a very limited choice of antibodies available. The specificity of the antibody used was confirmed by transfection of A375 cells with siRNA to the REV3L subunit (Additional file 1: Figure S5A). Detection of DNA Pol η (78 kDa) was more routine (Additional file 1: Figure S5B). Levels of DNA pol ζ increased in melanoma lines (A375, C32 and G361) in the 3 days following exposure to 3 μM cisplatin, but no increase occurred in MRC5v1 fibroblasts and PEO4 ovarian cancer cells. The largest increases (> 8-fold) were seen in A375 and C32 (see Fig. 7a for expression histograms and Additional file 1: Figure S6A for representative blots). All cell lines showed a small increase in DNA pol η expression (up to 2-fold) after cisplatin treatment, but this was not melanoma-specific (Fig. 7b and Additional file 1: Figure S6B). The remaining two melanoma lines (HBL and WM115 were not assayed for DNA polymerase levels.

To investigate the significance of increased DNA pol ζ levels in the response of A375 melanoma cells to cisplatin, we used siRNA to the catalytic subunit REV3L to knockdown DNA pol ζ and studied the effect on cisplatin treatment (Fig. 7c). The mean cell number of untreated control cultures increased 51-fold after 72 h. The mean cell number of 1 μM cisplatin-treated cultures was 67 ± 4% of the untreated control, and the mean cell number of the cisplatin-treated cultures transfected with scrambled siRNA was 48 ± 3% of the untreated control. While the mean cell number of the cisplatin-treated cultures transfected with DNA pol ζ siRNA was only 23 ± 1% of the untreated control, significantly 2-fold less than the scrambled siRNA control (*P* = 0.01, *n* = 4). Representative cell cycle profiles of control, cisplatin-treated, and cisplatin-treated cultures transfected with scrambled siRNA or DNA pol ζ siRNA after 72 h are shown in Fig. 7d. This time, compared to Fig. 6d, the small increase in the percentage of cells with sub-G1 DNA content (apoptotic) between cisplatin-treated and control cultures was just significant (*P* = 0.04 by Student’s t test, *n* = 4). However, the >2-fold increase in apoptosis between cisplatin-treated DNA pol ζ siRNA-transfected and scrambled siRNA-transfected control wells was highly significant (*P* = 0.007 by Student’s t test, *n* = 4). We conclude that the increase in DNA pol ζ constitutes an important part of the response to cisplatin in melanoma cells, resulting in improved survival and growth.

## Discussion

Cisplatin-induced DNA damage is repaired by a combination of nucleotide excision repair (NER) and homologous recombination repair (HRR). Standard chemotherapeutics, such as cisplatin, are ineffective on metastatic melanoma and we, and others, have shown that levels of a number of NER proteins in melanoma cells are elevated in response to cisplatin: ERCC1 and XPF [17], XPC and DDB2 [29], XPA (this publication). Such increases in DNA repair proteins likely contribute to the resistance of melanoma to chemotherapy. However, when we examined the key HRR protein, RAD51, we found that levels unexpectedly decreased by at least 65% in all five melanoma cell lines examined over a 3-day exposure to cisplatin, while levels in an ovarian cancer and a fibroblast cell line increased over the same period, as reported previously for lung cancer cell lines [24]. The RAD51 response of the melanoma lines was seen across a range of cisplatin concentrations up to 3 μM, which is cytotoxic for all melanoma and non-melanoma cell lines studied. The non-melanoma lines showed no reduction in RAD51 levels, even at the highest cisplatin concentration, showing that the difference in the RAD51 response of melanoma and non-melanoma lines to cisplatin is not simply a dose effect.

The RAD51 response of melanoma cells to cisplatin could have been due to decreased synthesis, or increased proteolysis, possibly simply as a consequence of cisplatin-induced apoptosis. Estimates of the half-life of RAD51 protein range from 5.5 to ~24 h in primary fibroblast and cancer cell lines, with no clear difference between cell types [30, 31]. We found no evidence for increased RAD51 proteolysis in cisplatin-treated cells via either of the two main pathways (proteasome and lysosome). Instead, decreased levels of RAD51 mRNA largely explained the reduction in RAD51 protein levels, suggesting that the RAD51 response to cisplatin most likely occurs at the transcriptional level. Evidence for the transcriptional specificity of this response was enhanced when a FLAG-tagged version of RAD51, expressed in melanoma cells at near endogenous levels, but under foreign promoter control, failed to show the cisplatin-induced reduction.

RAD51 is overexpressed in the majority of human cancers [32]. Oncoproteins, such as activated Ras and SV40 T antigen, stimulate the RAD51 promoter and RAD51 expression is positively regulated by the EGR1 transcription factor [32], while p53, which is mutated or functionally inactivated in the majority of cancers, binds to and represses the RAD51 promoter [33]. Transcription factor AP2 also binds to a site in the RAD51 promoter to repress transcription in a p53-dependent manner [34]. The E2F family of transcription factors complex with and are activated by hypophosphorylated forms of the Rb protein and other repressive pocket proteins, such as p130. E2F4/p130 complexes bind to a single site in the RAD51 promoter to repress transcription [35], while E2F1 binds to the same site to activate the promoter [36]. A variety of treatments, acting via some of these transcription factors, have previously been shown to repress RAD51 transcription: hypoxia [35]; methotrexate [37]; inhibition of PARP [38], HDACs [36], and tyrosine kinases such as Gleevec [39]. The melanoma-specific effect of cisplatin on RAD51 expression could also be mediated by one or more of the same transcription factors. In this regard we have previously shown that cisplatin regulates the MAPK pathway to induce increased expression of DNA repair gene ERCC1 and increase melanoma chemoresistance [17].

Flow cytometry revealed the ability of melanoma cells to continue cycling in the presence of 1 μM cisplatin. In A375 melanoma cells there was a weak G2 arrest at 24 h which had resolved by 48 h, while platinum sensitive (PEO1), resistant (PEO4) ovarian cancer cells and MRC5v1 fibroblasts all showed strong and persistent G2 arrests at 1 μM cisplatin. The failure of the majority of melanoma cell lines tested to show a G2 arrest following UV-irradiation has been reported previously [40].

How are melanoma cells able to continue to cycle in the presence of cisplatin-induced interstrand crosslinks (ICLs)? Our hypothesis, that the unexpected cisplatin-induced RAD51 reduction that we observed was important for this ability, was supported by expressing a FLAG-tagged version of RAD51 at endogenous levels under foreign promoter control. Levels of FLAG-RAD51 remained stable after cisplatin treatment, with cells expressing FLAG-RAD51 showing significantly reduced growth and increased apoptosis compared to cisplatin-treated control cultures during the three day time course of our assay. This conclusion was reached after correcting for the small negative effect that FLAG-RAD51 expression alone had on the growth of untreated cells. The use of an alternative inducible version of tagged RAD51, also expressing at endogenous levels, would avoid this complication.

Many of the helix-distorting ICLs caused by cisplatin are removed during G1 by a combination of the unhooking action of endonucleases, such as ERCC1-XPF, and translesion DNA synthesis (reviewed in [15]). This activity and the removal of cisplatin-induced intrastrand cross-links and monoadducts by NER could explain the increased levels of NER proteins we observed in cisplatin-treated melanoma cells. Specialised translesion synthesis (TLS) DNA polymerases provide a mechanism to allow cells to continue replication on a damaged DNA template, albeit at the expense of an increased mutation rate [26]. Complete bypass of cisplatin lesions requires two cooperating TLS DNA polymerases: DNA Pol η to insert dCTP opposite the 3′ guanine of an intrastrand crosslink (and presumably also opposite the G residue of an interstrand crosslink unhooked by ERCC1-XPF, or other endonuclease action), and DNA Pol ζ, which is more processive than DNA Pol η, for subsequent primer extension [27]. The majority of ICLs persisting into replication and encountered by replication forks are bypassed through a FANCM-dependent replication-traverse pathway to permit DNA synthesis to continue on the other side of the lesion [41]. The unrepaired ICLs are later dealt with by a post-replication repair process, likely to involve combined endonuclease action and translesion synthesis. While the minority of ICLs that block replication fork progression are unhooked by endonuclease action, generating a double strand break. Then, in a more protracted process, a combination of translesion synthesis, followed by RAD51-dependent HRR, enables replication to proceed [42].

In melanoma, as well as a range of other cancer and normal cell lines, increased Pol η expression was induced by exposure to chloroethyl nitrosoureas [43]. Like cisplatin, these agents generate ICLs, but these are between adjacent G and C residues, rather than adjacent Gs with platinum compounds. The Pol η transcriptional response was p53-dependent and increased levels of Pol η conferred increased resistance to the chloroethyl nitrosoureas. Although DNA Pol η also contributes to the tolerance of cisplatin adducts [26], DNA Pol ζ was found to be the major determinant in mediating platinum resistance in HeLa cells [44]. A 2-fold increase in DNA Pol ζ mRNA levels has been reported previously in a cisplatin-treated clonal derivative of the near-diploid, immortal human fibroblast line MSU-1.1 [45]. siRNA knockdown of the REV3L catalytic subunit of DNA Pol ζ in HeLa and MSU-1.1 cells resulted in increased sensitivity to cisplatin [44, 45]. Further support for the role of DNA Pol ζ in tolerance to crosslinks comes from the observation that knockouts for REV3L are hypersensitive to fotemustine, a crosslinking agent that has been used for therapy of malignant melanomas [46].

We found that levels of DNA Pol ζ were increased after cisplatin treatment by 2- to 10-fold in all three melanoma cell lines tested, but no increase was seen in ovarian cancer cells and fibroblasts. There was also an increase in DNA Pol η, but this was not melanoma-specific. The significance of the DNA Pol ζ result to the melanoma RAD51 response was demonstrated when we knocked down DNA Pol ζ levels and observed significantly slower growth and increased apoptosis after cisplatin treatment compared to the scrambled siRNA control during the three day time course of our assay. We have chosen to demonstrate the survival benefit to melanoma cells of their RAD51 response to cisplatin in a combination of short term growth, cell cycle analysis and apoptosis assays rather than conventional colony formation assays because we consider it more closely represents the in vivo situation. Patients typically receive platinum-based chemotherapy as a single intravenous infusion at three weekly intervals, with the maximum plasma concentration of free cisplatin in the range 1–5 μM decreasing rapidly with a half-life of less than 1 h [47], so therapeutic concentrations will only persist for less than 1 day at a time. Our assays, using cisplatin concentrations within the physiological range, show that the novel response, whereby levels of RAD51 are dramatically reduced, allows melanoma cells to survive and continue to grow under these conditions and that the compensatory increase in levels of DNA Pol ζ is an important part of this response. However, it is important to remember that, despite the RAD51 response, melanoma cells are under considerable stress during the exposure to these high concentrations of cisplatin and are growing more slowly with higher levels of apoptosis than untreated cultures and would likely not survive the non-physiological continuous cisplatin exposures needed for colony formation assays.

## Conclusion

We have shown that, rather than boosting levels of RAD51 on treatment with cisplatin, in a possibly unsuccessful attempt to repair high levels of ICLs encountered at stalled replication forks by the protracted HRR process, melanoma cells instead shut down HRR and boost levels of endonucleases, such as ERCC1-XPF, that can rapidly unhook crosslinks encountered prior to, and possibly also during replication but before replication fork arrest, and then use translesion synthesis DNA polymerases to allow replication to proceed. In addition, elevated levels of endonucleases and translesion synthesis DNA polymerases can be used to boost post-replication repair of ICLs bypassed by replication traverse. This approach of DNA damage tolerance rather than immediate repair may be a more appropriate strategy for an aggressive and rapidly growing tumour like melanoma, that also enables it to better survive exposure to platinum chemotherapeutics. However, this strategy could also result in melanoma exposing an Achilles heel, a BRCAness phenotype rendering it more susceptible to PARP inhibitor therapy.

## Additional file

Additional file 1:
**Figure S1.** Western blots showing that the reduction in RAD51 levels after cisplatin treatment in melanoma cells is not seen in other cell types. Blots support the RAD51 quantification shown in Fig. 2 of the main text. **Figure S2. ** Images showing that RAD51 repair foci are not induced by short term cisplatin treatment of melanoma cells. **Figure S3. ** The cisplatin-induced reduction in RAD51 levels in melanoma cells is not affected by proteasome or lysosome inhibitor treatment. **Figure S4. ** Melanoma cells show only a weak cisplatin-induced G2 arrest. **Figure S5.** Specificity of antibodies to translesion synthesis DNA Polymerases. **Figure S6. ** Western blots showing increased levels of DNA Polymerase zeta in cisplatin-treated melanoma cells. Blots support the DNA Pol ζ and Pol η expression data shown in Fig. 7 of the main text. (PDF 2393 kb)

## Abbreviations

BRAF: v-Raf murine sarcoma viral oncogene homologue B
BRCA: Breast cancer susceptibility gene
CDDP: Cisplatin
DAPI: 4′,6-Diamidino-2-phenylindole
DMEM: Dulbecco’s modified Eagle’s medium
DMSO: Dimethyl sulfoxide
DNA pol ζ: DNA Polymerase zeta
DNA pol η: DNA Polymerase eta
ERCC: Excision repair cross-complementing gene
FCS: Foetal calf serum
HRP: Horseradish peroxidase
HRR: Homologous recombination repair
ICLs: Interstrand crosslinks
MAPK: Mitogen-activated protein kinase
NER: Nucleotide excision repair
PARP: Poly (ADP-ribose) polymerase
RAD: Radiation repair gene
REV3L: Catalytic subunit of DNA Polymerase zeta ζ
RPMI: Roswell Park Memorial Institute
RT-PCR: Reverse transcription polymerase chain reaction
siRNA: Small inhibitory RNA
SRB: Sulphorhodamine B
TLS: Translesion synthesis
XPA: Xeroderma pigmentosum complementation group A
XPF: Xeroderma pigmentosum complementation group F
