# Supplementary material for: Melanoma cells replicate through chemotherapy by reducing levels of key homologous recombination protein RAD51 and increasing expression of translesion synthesis DNA polymerase ζ
Source: BMC Cancer. 2017 Dec 18;17:864. doi: 10.1186/s12885-017-3864-6 (PMC5735618; doi:10.1186/s12885-017-3864-6)
Supplement: Additional file 1: — Figure S1. Western blots showing that the reduction in RAD51 levels after cisplatin treatment in melanoma cells is not seen in other cell types. Blots support the RAD51 quantification shown in Fig. 2 of the main text. Figure S2. Images showing that RAD51 repair foci are not induced by short term cisplatin treatment of melanoma cells. Figure S3. The cisplatin-induced reduction in RAD51 levels in melanoma cells is not affected by proteasome or lysosome inhibitor treatment. Figure S4. Melanoma cells show only a weak cisplatin-induced G2 arrest. Figure S5. Specificity of antibodies to translesion synthesis DNA Polymerases. Figure S6. Western blots showing increased levels of DNA Polymerase zeta in cisplatin-treated melanoma cells. Blots support the DNA Pol ζ and Pol η expression data shown in Fig. 7 of the main text. (PDF 2393 kb) [file 12885_2017_3864_MOESM1_ESM.pdf]

ADDITIONAL FILE

Figure S1. Western blots showing that the reduction in RAD51 levels after cisplatin treatment in melanoma cells is not seen in other cell types. Blots support the RAD51 quantification shown in Figure 2 of the main text. Five human melanoma cell lines (A375, C32, G361, HBL and WM115), two human ovarian cancer cell lines (PEO1 and PEO4) and the immortalised human fibroblast line (MRC5v1) were untreated, or treated with 0.3, 1 or 3 µM cisplatin for 24 (Day 1), 48 (Day 2) or 72 h (Day 3) and western blotted for RAD51. β-actin served as the loading control.

Figure S2. Images showing that RAD51 repair foci are not induced by short term cisplatin treatment of melanoma cells. Control A375 melanoma, MRC5v1 immortalised fibroblasts and PEO4 ovarian cancer cells growing on coverslips and cells treated for 24 h with 6 µM cisplatin were fixed and stained. Immunofluorescence was used to detect RAD51 foci (green) and DAPI staining (blue) was used to visualise nuclei. Images support the quantification of RAD51 DNA repair foci shown in Figure 3 of the main text.

Figure S3. The cisplatin-induced reduction in RAD51 levels in melanoma cells is not affected by proteasome or lysosome inhibitor treatment. (A) Total protein extracts from control A375 and C32 human melanoma cells, cells treated with 3 µM cisplatin (CDDP) alone for 72 h, and combined with 1 µM proteasome inhibitor MG132, were western blotted for RAD51. β-actin served as the loading control. (B) Total protein extracts from control A375 cells and cells treated with 1 µM proteasome inhibitor MG132 for 24 h were western blotted for ERCC1 as a positive control for the effect of proteasome inhibitor treatment. (C) Total protein extracts from control A375 cells, cells treated for 72 h with 3 µM cisplatin (CDDP) alone, 10 nM proteasome inhibitor bortezomib alone, and combined cisplatin and bortezomib were western blotted for RAD51. (D) Total protein extracts from control G361 melanoma cells, cells treated for 72 h with 3 µM cisplatin (CDDP) alone and combined with 1 µM lysosome inhibitor Bafilomycin A1 were western blotted for RAD51.

Figure S4. Melanoma cells show only a weak cisplatin-induced G2 arrest. Cell cycle status of melanoma (A375), ovarian cancer (PEO1 and PEO4) and immortalised fibroblast (MRC5v1) cells by flow cytometry. Untreated cells, or cells treated with cisplatin, were fixed and stained with propidium iodide to display their DNA content and so their cell cycle status. Intensity of propidium iodide staining on the x axis, frequency on the y axis. For each treatment the percentage of cells with <2n, G1, S, G2/M, and >4n DNA contents, determined by FlowJo cell cycle analysis software (Ashland ,OR), is indicated across the top of the plots. (A) Control cultures. (B) Cultures treated with 0.3 or 1 µM cisplatin for 24 or 48 h.

Figure S5. Specificity of antibodies to translesion synthesis DNA Polymerases. (A) DNA Pol zeta. Western blot of protein extracts from A375 cells made 72 h after transfection with control or DNA Pol ζ siRNA. Note the lack of DNA Pol ζ protein (353 kDa) in the DNA Pol ζ siRNA-transfected sample. The blot was reprobed for the nuclear markers Lamins A and C (74 and 65 kDa, respectively) as a loading control. The positions of molecular

weight markers (in kDa) are shown. (B) DNA Pol η. Western blot of protein extracts from A375 control (Cont.) cells and cells treated for 24 h with 3 µM cisplatin (CDDP). The main DNA Pol η band at 78 kDa is indicated.

Figure S6. Western blots showing increased levels of DNA Polymerase zeta in cisplatin-treated melanoma cells. Blots support the DNA Pol ζ and Pol η expression data shown in Figure 7 of the main text. Melanoma cell lines (A375, C32, G361), ovarian cancer cell line, PEO4, and immortalised fibroblast line, MRC5v1, were untreated, or treated with 3 µM cisplatin for 24, 48 or 72 h and western blotted for translesion synthesis DNA Polymerases. (A) DNA Pol ζ. (B) DNA Pol η. β-actin served as the loading control.

Figure S1

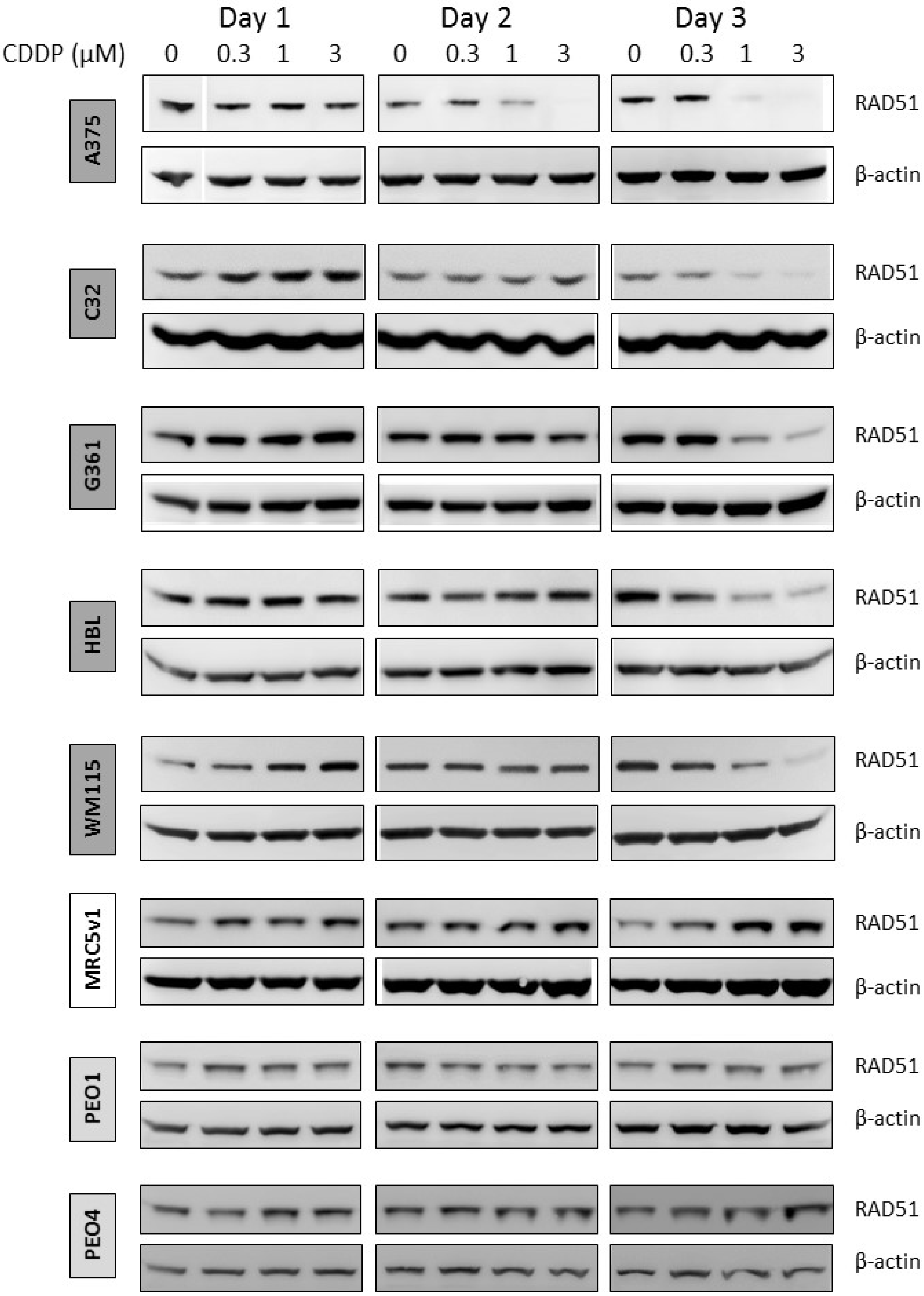

Day 1
Day 2
Day 3
CDDP (μM)
0
0.3
1
3
A375
C32
G361
HBL
WM115
MRC5v1
PEO1
PEO4
RAD51
β-actin

Figure S2

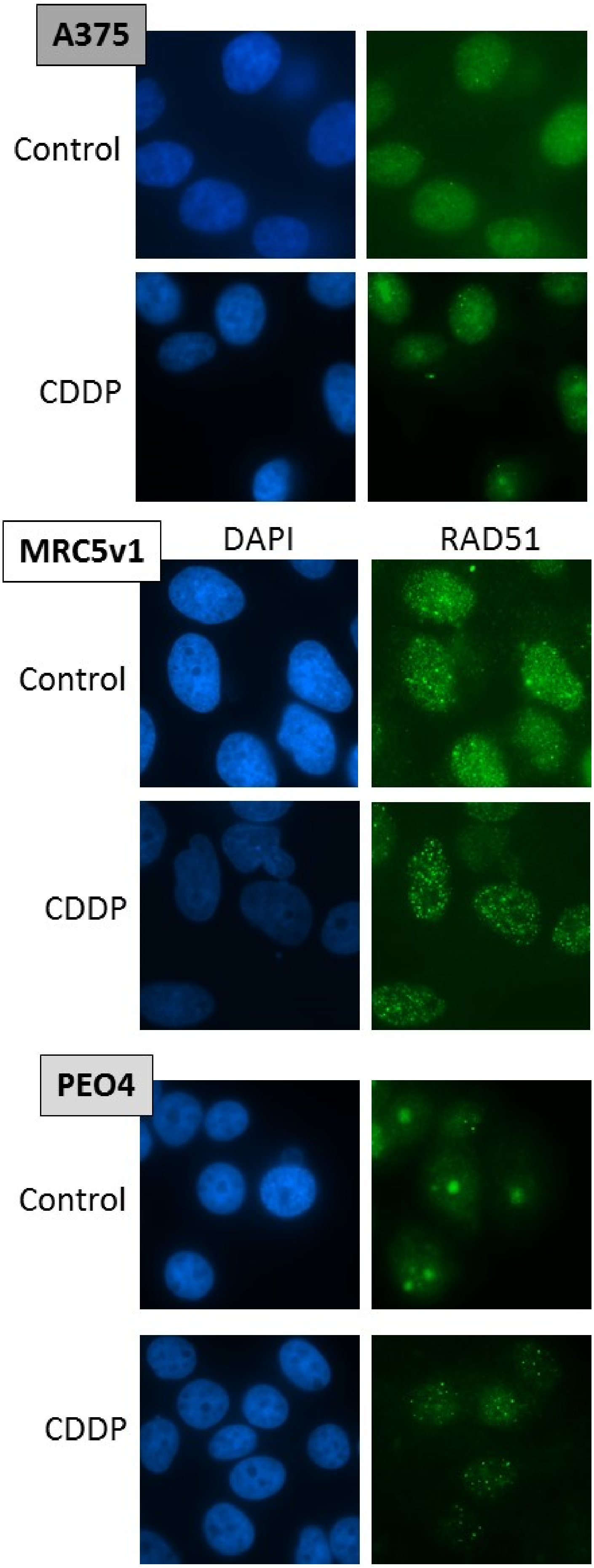

A375
Control
CDDP
MRC5v1
DAPI
RAD51
Control
CDDP
PEO4
Control
CDDP

Figure S3

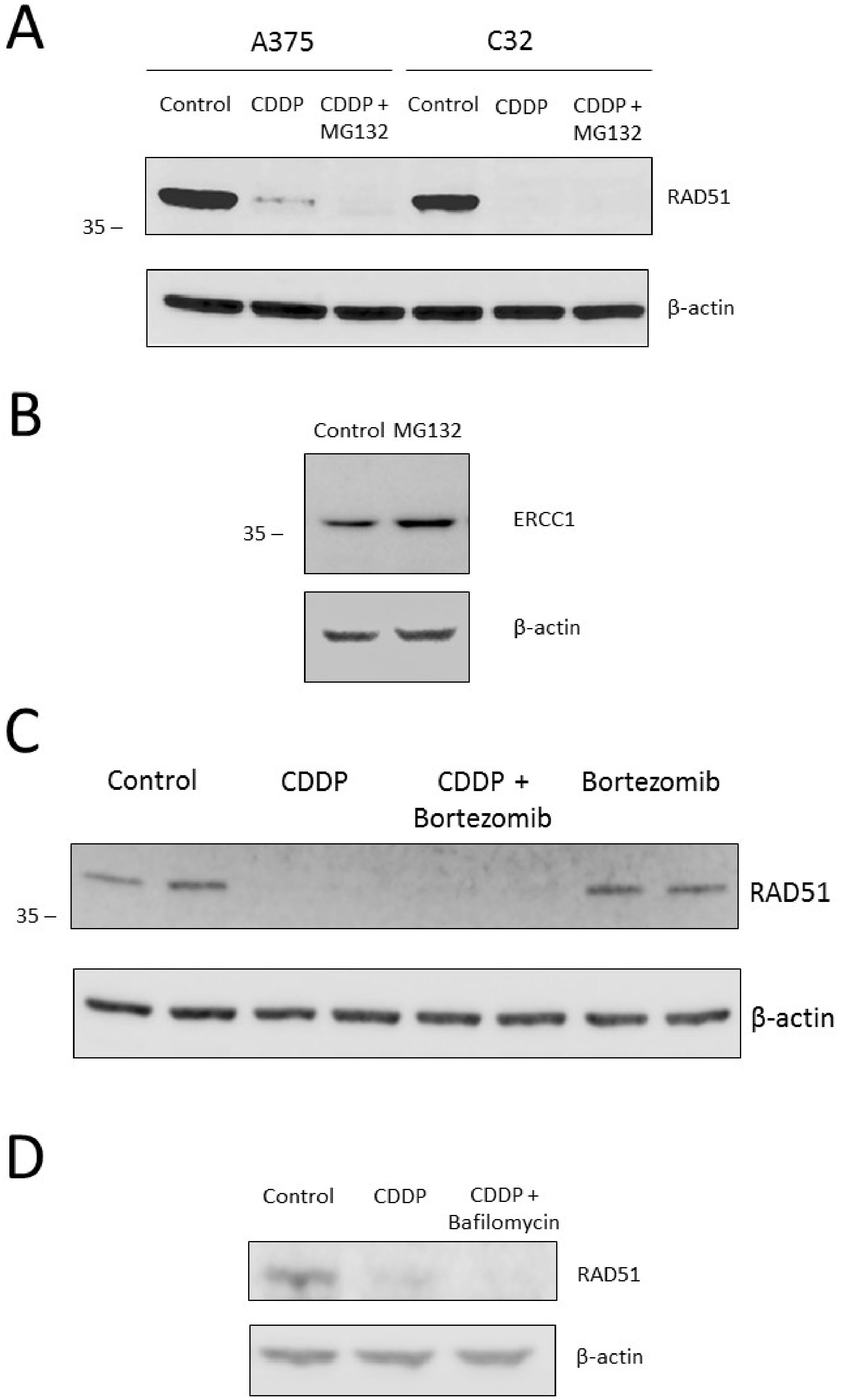
A
A375
C32
Control
CDDP
CDDP + MG132
Control
CDDP
CDDP + MG132
35 –
RAD51
β-actin
B
Control MG132
35 –
ERCC1
β-actin
C
Control
CDDP
CDDP + Bortezomib
Bortezomib
35 –
RAD51
β-actin
D
Control
CDDP
CDDP + Bafilomycin
RAD51
β-actin

Figure S4

A Control cultures

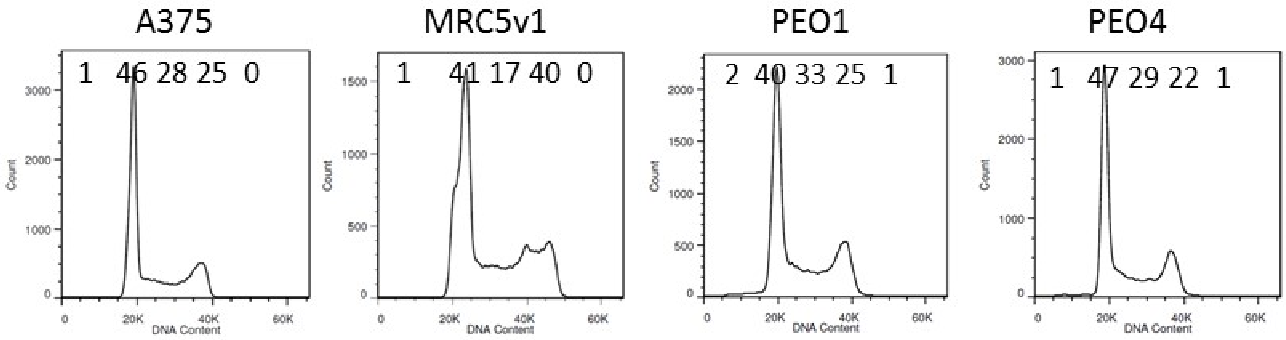

B Cisplatin-treated cultures

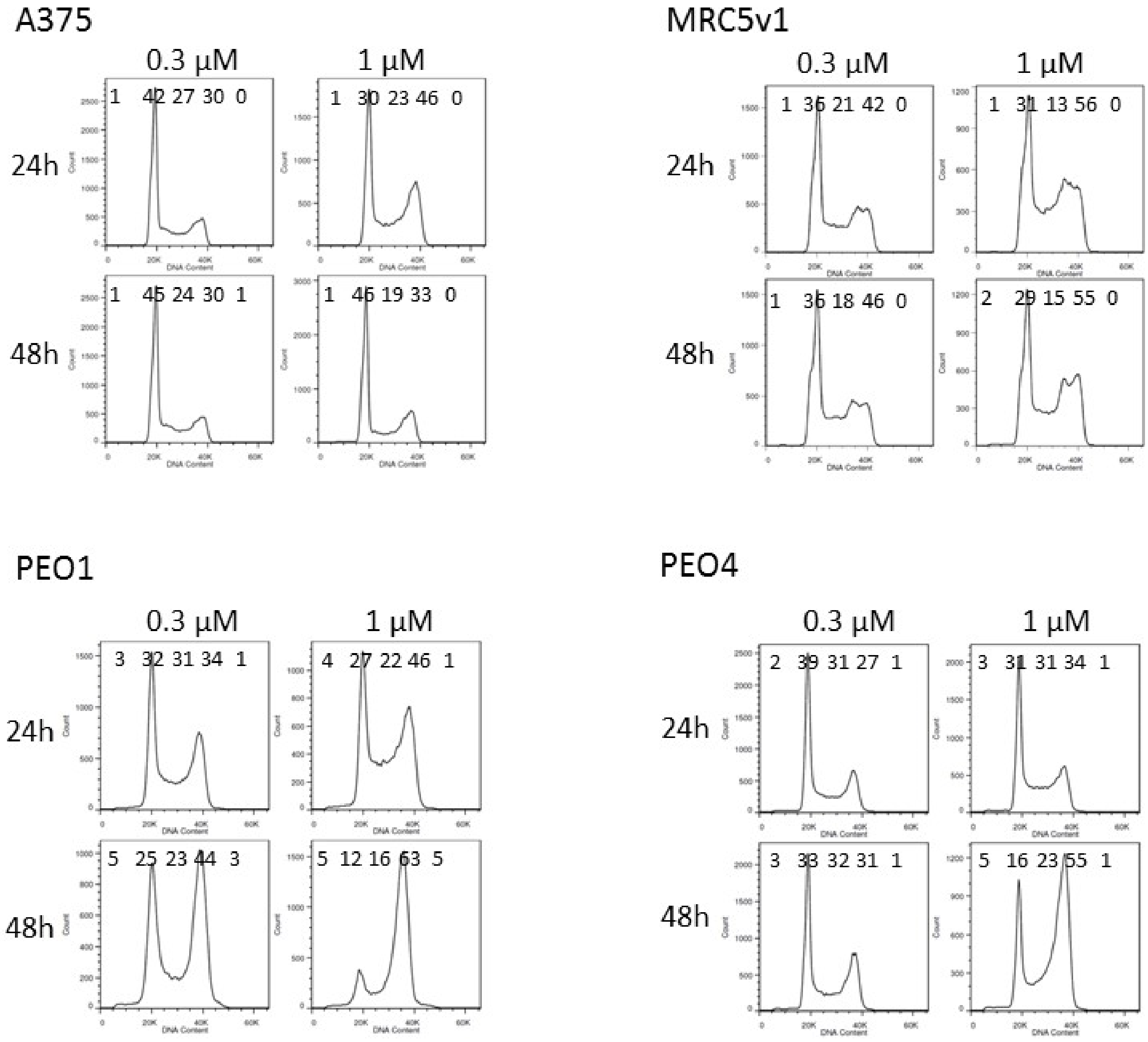

Figure S5

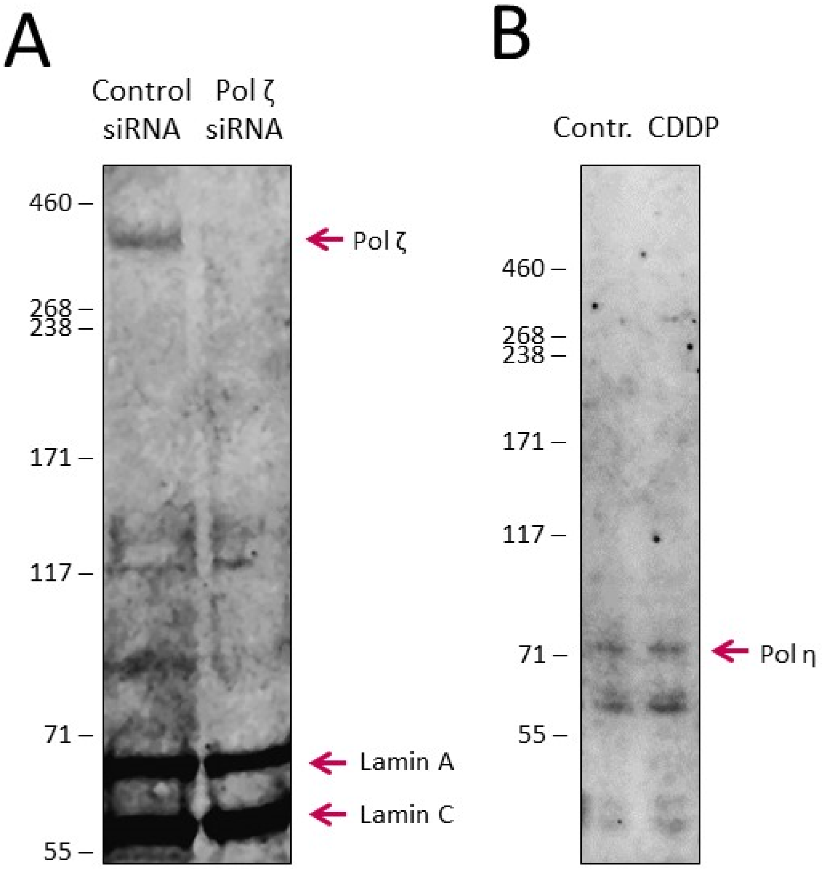
A
Control siRNA
Pol ζ siRNA
460 –
268 –
238 –
171 –
117 –
71 –
55 –
Pol ζ
Lamin A
Lamin C
B
Contr.
CDDP
460 –
268 –
238 –
171 –
117 –
71 –
55 –
Pol η

Figure S6

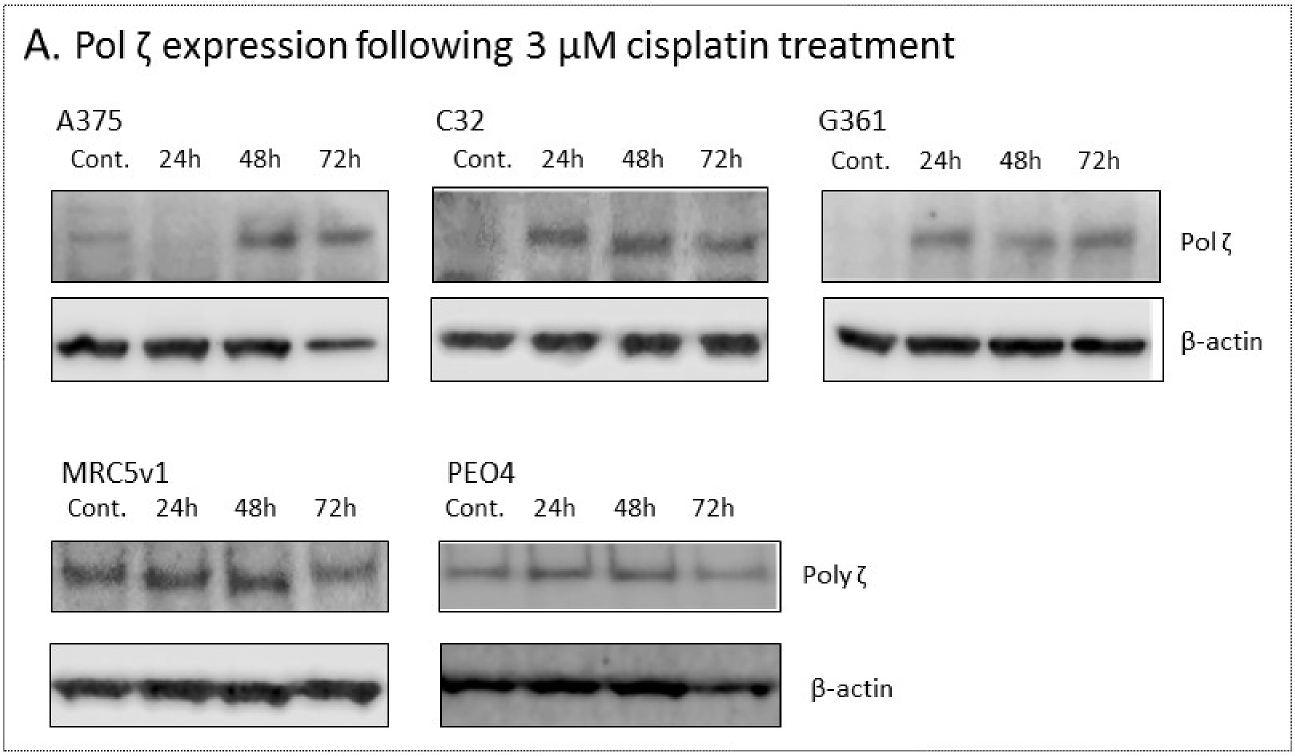
A. Pol ζ expression following 3 µM cisplatin treatment
A375
Cont. 24h 48h 72h
C32
Cont. 24h 48h 72h
G361
Cont. 24h 48h 72h
Pol ζ
β-actin
MRC5v1
Cont. 24h 48h 72h
PEO4
Cont. 24h 48h 72h
Poly ζ
β-actin

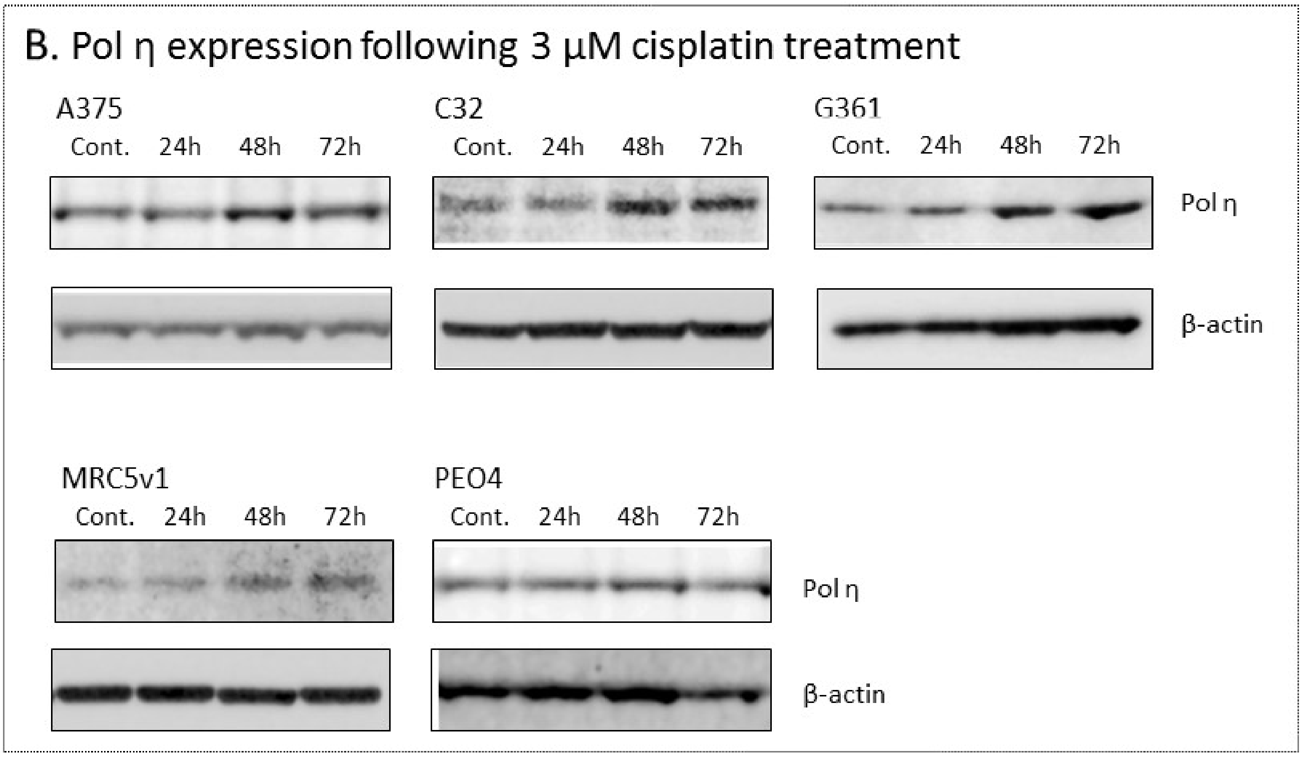
B. Pol η expression following 3 µM cisplatin treatment
A375
Cont. 24h 48h 72h
C32
Cont. 24h 48h 72h
G361
Cont. 24h 48h 72h
Pol η
β-actin
MRC5v1
Cont. 24h 48h 72h
PEO4
Cont. 24h 48h 72h
Pol η
β-actin
